# The Nutritional Condition of the Spanish Soldier: “*Spain. Nutrition Survey of the Armed Forces, a Report by the Interdepartmental Committee on Nutrition for National Defence 1958*”

**DOI:** 10.3390/ijerph182312623

**Published:** 2021-11-30

**Authors:** Pedro Fatjó Gómez, Francisco Muñoz Pradas, Roser Nicolau Nos

**Affiliations:** 1 Department of Economics and Economic History, Autonomous University of Barcelona, 08193 Bellaterra, Spain; Pedro.Fatjo@uab.cat (P.F.G.); Roser.Nicolau@uab.cat (R.N.N.); 2 Department of Geography, Autonomous University of Barcelona, 08193 Bellaterra, Spain

**Keywords:** health and nutritional history, military nutrition, malnutrition-related diseases, Spanish male population

## Abstract

The study of the nutritional transition in Spain must combine sources concerning the health conditions and the nutritional profile of the population. Such an approximation to the issue is, as a rule, not possible until the two final decades of the 20th century. However, the report on the nutritional status of the Spanish army, undertaken by the American Interdepartmental Committee on Nutrition for National Defence (ICNND) in 1958, combines both approaches. The report is based on the medical examination of 10727 army drafts. First, the article contextualised the report’s sample geographically and demographically; second, it validated the variables used statistically; and third, it explored the relationship between the diseases diagnosed, the biomarkers yielded by blood and urine tests, and the diet. The main results were as follows: (a) the report confirmed that the military population under examination did not suffer from severe dietary shortcomings; (b) the sample presents a double bias, geographical (overrepresentation of southern provinces) and institutional (underrepresentation of the land forces).

## 1. Introduction

The study of the evolution of, and mutual relationship between, health and nutritional standards in Spain is conditioned by the uneven nature and chronology of the sources. Regular health standards surveys promoted by the WHO did not begin in Spain until the 1980s [1] (pp. 110–111). For earlier periods, the most common sources concern diseases of compulsory declaration and hospital records. These can be valuable to establish provincial morbidity statistics but cannot be used to infer nutritional deficiencies. As such, it seems that the assessment of the direct effect of nutritional deficiencies on health standards can only be approached through cause of death statistics, which, after 1900, adopted the International Classification of Diseases (CIE), reflecting a series of diseases that are directly related to malnutrition [1] (pp. 108–110) [2]. Concerning the population’s alimentary habits, the available statistics have conditioned the two most usual approaches. Historical series of food production and foreign trade enable us to estimate apparent consumption, while household budget surveys present information on the quantities of food consumed and their prices. These surveys record information not only about the monetary cost of food but also the actual amounts consumed. In Spain, these surveys began in the late 1950s and continued being produced regularly in the following decades [3]. The data collected were used in a number of studies about the nutritional value of Spanish diets [4,5]. In the context of the problematic development of rural areas in the 1960s, specific surveys were undertaken to assess nutritional standards in the Spanish countryside. At the same time, the launching of a program of nutritional education (*Edalnu*) allowed the collection not only of dietary data, but also of clinical and anthropometric information in relation to sanitary and socio-economic variables [6]. Our knowledge about the relationship between nutritional deficiencies and health standards, with the exception of a few studies undertaken in Madrid during the Civil War and its immediate aftermath [7], is limited to the examination of military drafts records [8]. This assumes adopting stature as the expression of the net balance between favourable and unfavourable factors in the physical development of children and adolescents. However, the complexity of the process severely questions the reliability of the inferences that this approach necessarily demands. With this background, the survey published in 1958, “Spain: Nutrition Survey of the Armed Forces” (SNSAF)—on the initiative of the Interdepartmental Committee on Nutrition for National Defence (ICNND) of the USA—on a sample of Spanish soldiers, is a unique source. The use of the adjective ‘unique’ is entirely justified by the very nature of the study, which explicitly aimed to assess the nutritional standard of Spanish troops through various medical and dietary examinations and explore the relationship between nutritional and health standards. This survey has gone almost totally unnoticed until recently [9], and has, therefore, not been taken into account in the analysis of the Spanish nutritional transition.

This paper aims to analyse this survey and examine the published results. Although its results were conditioned by a series of factors that will be explained below, the study is a unique opportunity to shed some light on the biomedical condition of Spanish young males in the 1950s. In this period, a series of political and economic processes put Spain on the path of the rapid economic development, undergone in the 1960s, and closed a period of progressive deterioration of living standards caused by the Civil War [10]. Moreover, it must be remembered that this population was born and grew up during the war and post-war period, particularly during the so-called “hunger years (1940–1952)” [11].

The text is divided into several sections. First, the document is presented, and its origins, content, and the methodology used are described. Second, it presents its target sample, the results of the clinical examinations undertaken and, finally, the composition of the diets. The third section discusses one major contribution of the survey, namely the relationship between the intake of a number of micronutrients and the incidence of diseases related to nutritional deficiencies. The final section assesses the concluding remarks of the authors of the report and, especially, its representativeness as a source for the study of the biomedical standards of the Spanish population in the 1950s.

## 2. Materials and Methods

### 2.1. Origin, Objectives, and Methods of the Survey

The ICNND was officially created in 1955, with the participation of several state departments, including home defence; the medical sections of the army, navy, and air force; specialised agencies dealing with international cooperation; education in healthcare and wellbeing; agriculture; the Atomic Energy Commission; and international agencies, such as the WHO, the FAO, the Pan American Health Organization, UNICEF, and several American universities [12]. The aim of this body was to investigate and provide technical assistance to the armed forces, and the civilian population, of the USA’s allies in the fields of nutrition and healthcare. This crystallised in a series of reports on the healthcare and nutritional status of the armed forces, and sometimes also the civilian population, of approximately 30 countries between 1955 and 1971.

In addition to undertaking these healthcare and nutritional studies, the program also trained local personnel in the following: nutrition evaluation methods; chemical and biochemical analysis; diet design and food processing; the setting up of nutrition laboratories; and the general improvement of local nutritional standards, based on locally available resources [13].

The study undertaken in Spain took place between April and June 1958. The American team included 9 people (a medical lieutenant colonel, a cardiologist, 2 nutritionists, 3 biochemists, a statistician, and a food and agriculture expert). The Spanish team was constituted by 22 officers (with 10 military doctors, 5 military pharmacists, a veterinary major, a logistics major, and a nurse lieutenant). The team also included 15 NCOs (5 brigadiers, 2 sanitary brigadiers, 2 pharmacist brigadiers, 1 pharmacy assistant, 3 nurse brigadiers, 1 nurse sergeant, and 1 clerk) [14] (pp. I–IV).

The main aim of the study, as with all the reports issued by the ICNND, was to evaluate the nutritional standards among the troops and to obtain precise data about their overall health standards. Although the characteristics of the sample and the results of the medical checks will be examined in more detail below, it must now be stated that the sample comprised 10,727 soldiers. All participants underwent a general medical check (‘*Brief examinations*’) and 2145 underwent a more detailed check (‘*Detailed examinations*’); of these, 520 blood and urine samples were taken (‘*Biochemical examinations*’). These examinations were followed by an analysis of the nutritional value of the diets consumed in different military facilities.

The content of the document, which is 106 pages long, including 40 tables, is summarised in Table 1. Both types of medical exam are given the same number of tables (5), but the number of diseases diagnosed varies. The biochemical exams are presented in a larger number of tables (7), especially because relations between certain indicators and diseases related to malnutrition are explored. As such, 17 tables were used to present the main findings of the clinical exams undertaken. The second group of tables (19 in total) presents the study of the diets. The central block of tables addresses the nutritional composition of the meals served in various military facilities. Some of the information thus presented is compared with known data for the whole of the Spanish population for that time.

The report had important repercussions. One year after the publication of the document, in 1959, the journal *Ejército de Tierra Español* published the first of three articles signed by Medical Lt Colonel Trigueros Peñalver, of the *Jefatura de Servicios de Sanidad Militar*, a member of the team that produced the ICNND report [15]. Soon after, in 1960, the General Staff logistics Major Martínez Marañón published a book about logistic services in the American Army; the final chapter of which included several recommendations to improve organization and logistics that were partially based on the sections of the ICNND that dealt with military food supply and nutrition [16]. It is also highly significant that it fell to Spain to organise the ICNND’s fifth international conference in 1962 [17]. In this way, albeit slowly, institutional changes were introduced in order to improve and modernise military food supply services The *Departamento de Nutrición de las Fuerzas Armadas* was created in 1966, and its organisational structure was fully established by 1967 [18]. The final step in this process was the publication in 1968 of the first food manual for the armed forces, which all three branches (army, navy, and air force) had the obligation to adopt [19].

### 2.2. Data and Methodology

As noted, the study comprised 10,727 Spanish soldiers. The data collected included height, weight, and symptoms, should any of the 14 diseases related to malnutrition be diagnosed. Biographical data, including age, time in the service, place of birth, and father’s occupation, were also recorded. Out of the initial sample, 2145 individuals underwent a more detailed medical exam, so as to detect symptoms for a list of 52 diseases. In the tables, these diseases were divided into ten groups, based largely on anatomic criteria, except for the entry ‘infectious diseases’, which included malaria, brucellosis, and typhus. The list and groups created for both examinations are presented in Appendix A Table A1. This second medical examination was followed by biochemical blood and urine tests with a sample of 520 soldiers. Blood tests involved the measurement of nine indicators (plasma protein, haemoglobin, haematocrit, vitamin A, carotene, cholesterol, vitamin C, and riboflavin) and three in the urine tests (thiamine, riboflavin, and methyl nicotinamide). The aim of these tests was to complement the symptomatology of diseases related to malnutrition with biochemical evidence. These results were later compared with the study of the nutritional value of mess rations. In order to estimate the intake of energy and nutrients, food was weighed before and after cooking, and waste and leftovers were discounted. Nutrient estimates were based on the food composition tables published by FAO, although for some items the data used was that published by the American Department of Agriculture. In addition to the rations served in the mess halls, the study also took into account the consumption of food in military canteens, even if this came out of the soldiers’ pockets.

The data was computer-processed (through the use of punched cards), which accounts for the variety and systematic nature of the tables published with the report. The most disaggregated data level corresponds to each of the military facilities included in the study. The variables used to process the information in these double-entry tables include the military branch (army, navy, or air force), time in the service (divided into three or four intervals), residence (urban or rural), the recruit’s father’s occupation (farmer, craftsman, professional, and others), distribution according to standardised weight intervals (using those used to classify the American adult male population), and distribution by examiner. This study has taken the first four of these variables, which are considered the most relevant.

In order to facilitate the statistical analysis of the data by increasing the number of observations per cell, we have worked on the basis of the groups of diseases presented in Appendix A Table A1. Concerning methodology, the usual statistical variables have been calculated, including variable dependence tests for contingency tables, such as Pearson’s chi-square and G^2^ maximum likelihood tests. In addition, Cramer’s V was used to assess the association between variables and the disaggregation of test statistic G^2^, in order to define the range of the independence test for each of the groups of diagnosed diseases.

In relation to the analysis to be undertaken in this study, it is worth keeping in mind the nature of the data published in the report; that is, the object of study is merely the tables created for the report. The implications of this are twofold: first, the variables and statistical values concerning the biochemical condition of the sample reflect average values (and standard deviations when appropriate) with reference to the tabulation variables. In the study of relations in terms of territorial units of reference, such as military facilities, military regions, and place of residence, an ‘ecological correlation analysis’ perspective is adopted; as such, the results cannot be re-scaled to individual terms of analysis. The second implication has to do with a ‘composition effect’, which is the direct result of the way in which the tables were constructed. In this way, the incidence of disease can be distributed between the different armed forces branches and then compared. It must be observed that, if these comparisons are made, the age, place of origin, socio-economic status, and time in the service variables must be excluded from the analysis. As we shall see in the following section, recruitment age, medical access criteria, and socio-economic status of recruits varied from branch to branch. Therefore, there were differences in the selection of recruits, and this affects the comparative exercise. Without more precise data or more disaggregated tables with which to ‘standardise’ the indicators, this limitation is difficult to overcome. These results must be read taking this circumstance into account.

## 3. Results

### 3.1. Analysis of the Surveyed Population Subsection

Our examination of the characteristics of the sample will focus on three aspects: the first is related to the size of the sample; the second, to its geographical and military distribution; and the third, to its internal composition, according to several variables.

The report states that the size of the sample (10,727 soldiers) and the selection of the target military facilities depended on logistic and opportunity criteria (the study was to be carried out within a deadline of three months). No statistical sampling protocol was thus applied. The survey comprised approximately 7% of the total number of recruits for 1958, estimated at 149,855 [20]. Based on the associated estimated error margins $E=1.96\left(\frac{\sigma }{\sqrt{n}}\right)$ [21] and a 5% confidence interval (normal distribution), this proportion can be regarded as significant. For instance, concerning height and weight, standard deviations calculated on the basis of the statistics published in the *Anuario Estadistico Militar* (Military Statistics Report) for 1962 (height: 6.28 cm; weight: 6.96 kg) yielded 0.10% and 0.25% average estimate errors for these variables, respectively; that is, 16 millimetres and 157 g. However, as noted, the two subsequent medical exams involved a smaller number of recruits. In these, the margin of error must increase with the decrease in the size of the sample. Unfortunately, in these cases the standard deviations are not available.

**Figure 1:**
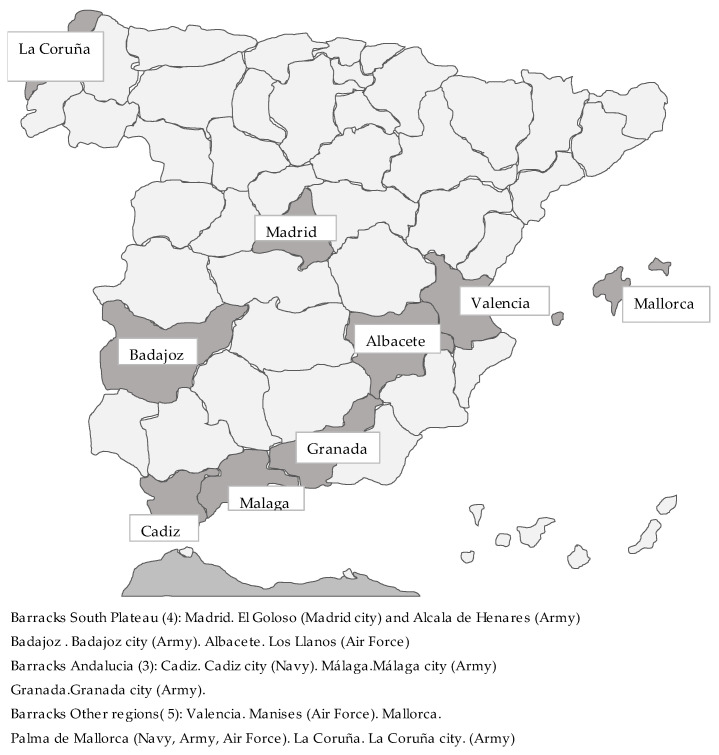
Locations of the military units surveyed.

Figure 1 shows the provinces in which the ten locations and twelve facilities included in the survey were located, and also indicates the branch to which each facility belonged. The provinces are geographically grouped into three regions, which in the report are referred to as ‘South-plateau’, ‘Andalusia’, and ‘Other regions’. The map thus illustrates the geographical concentration of the facilities chosen for the study. As noted, the authors of the report explained this as the consequence of the practical difficulties involved in incorporating other regions to the study. With no data about the distribution of the armed forces within each region, it is virtually impossible to evaluate the magnitude of the discrepancy between this distribution and that of the sample. In other words, this discrepancy would be greater had the armed forces been deployed mostly in the north and smaller if they were, like the sample, primarily deployed in the southeast. On the other hand, military needs would require recruits being deployed in regions different from those in which they resided. Although the tables published do not allow us to make a detailed analysis between location of deployment and location of origin, they offer some data (see Table 2) that can be used to reach some preliminary conclusions.

A total of 40% of the 10,727 subjects were deployed in the provinces of Madrid, Badajoz, and Albacete, while the remaining 60% were deployed, in fairly similar proportions, in barracks located in 3 Andalusian provinces and the provinces of Valencia, Mallorca, and La Coruña. However, only 57% of the soldiers that took part in the study were born in the province in which they were deployed, while the remaining 43% were born in other provinces.

**Figure 2:**
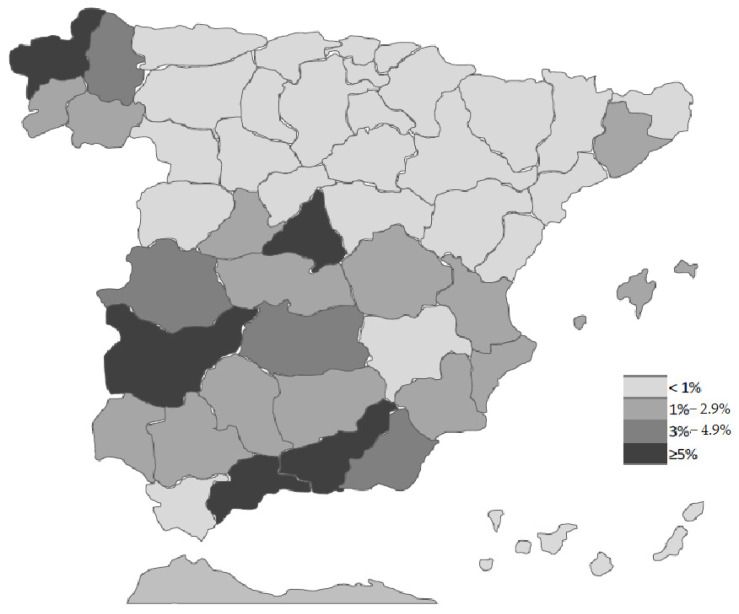
Soldiers surveyed by province of origin (percentage) (N = 10,727).

**Figure 3:**
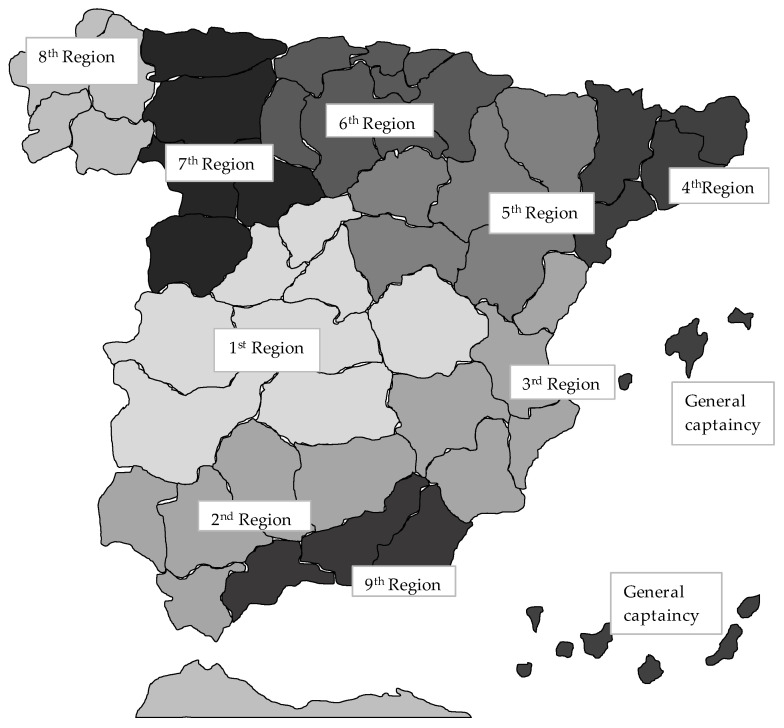
Military regions in Spain (1958). (Army).

Figure 2 presents the province of birth of the survey’s participants, showing that there were participants from all provinces. However, most participants were fairly local, and the northern regions of the country are underrepresented. Figure 3 and Table 3 illustrate that the military regions were unevenly represented in the sample. Regions 1, 8, and especially 9 are overrepresented, while the northern regions 4, 5, 6, and 7 are significantly underrepresented.

The representativeness of the sample does not only depend on its geographical distribution, but also on its military composition. This is relevant, because legal drafting conditions and physical and health selection criteria of recruits were not the same in all armed forces branches.

Following the new 1940 military act [23], followed by the 1943 regulations [24] (in force until 1968 and 1969 respectively), army and air force recruits in any given year (they joined the ranks in January) were those men who had turned 21 between 1 January and 31 December in the previous year. The study subjects were, therefore, between the ages of 21 and 23, as the duration of the military service was between 18 and 24 months. The navy, on the other hand, had its own act and regulations, passed with the Republican military laws of 1933 and 1935 [25] (a few minor changes were introduced in 1941 and 1942). These were in force until 1968–9. The base of navy recruits was the so-called *Inscripción Marítima*, which listed all Spaniards active in the maritime, fishing, and offshore industries. They were drafted when they turned 19 but could request joining voluntarily 1 year earlier, so they effectively joined soon after turning 20 or at the age of 19, depending on the month of birth. The age bracket was, therefore, 19–21.

Army and air force recruits were subject to the same health-related total and temporary causes of exclusion—a list of 208 diseases and physical limitations [24] (pp. 141–154). The list for the navy was 253 diseases, which implies a more rigorous selection than in the other 2 branches [25] (pp. 45–54). On the other hand, the air force attracted a larger proportion of volunteers, and had its own boards to examine physical and health conditions [26]. Table 4 compares the distribution of recruits in the 3 branches for the 1967 draft, the 1st year for which this information is available. Navy and Air Force recruits are overrepresented in the sample.

Table 5 presents other characteristics of SNSAF subjects. Soldiers that came from households in which self-consumption was above 50% of total consumption were regarded to come from a rural background; 69% of the sample met this criterion, and the proportion of subjects whose father worked in agriculture was 58%. The proportions were significantly lower in the air force and the navy, owing to the fact that the presence of recruits from the ‘South plateau’ region, where the greatest number of rural recruits whose fathers worked in agriculture came from, was negligible in these branches.

Finally, the table presents the average height and weight of the subjects in the sample. Air force recruits were, on average, approximately 2 cm taller and 3 kg heavier than the recruits in the other two branches. Most of the differences attested per region and branch (Table 6) are statistically significant.

In conclusion, the sample presents a double bias: geographical and institutional. The south and centre of the Iberian Peninsula and the air force and navy are overrepresented. The latter group was, obviously, particularly numerous in coastal provinces (seven out of the ten). This bias in favour of air force and navy recruits, who were better fed owing to their geographical origin and the stricter entry requirements, is probably offset, at least partially, by the overrepresentation of drafts from the central and southern regions, which throughout the first half of the 20th century presented worse living condition indicators than the northern regions, for instance, infant mortality [27] (pp. 44–58), income distribution [28], per capita income [29], and milk consumption [30]. This can be illustrated by the average height of the subjects of the study, which was 164.6 cm, compared with 166.21 cm for the whole draft, according to the Military Statistics Report [20]. This 2 cm difference shows that the under-representation of barracks located in the north of Spain, particularly the Basque Country and Catalonia, regions with historically greater heights than in the south [31], has a greater impact on this indicator than the over-representation of the air force in the sample.

### 3.2. Clinical and Biochemical Examinations Results

The differences between the two medical examinations went beyond the level of detail, and also included what the authors of the report referred to as ‘diagnosis effect’. This concept subsumes the effect of the adoption of different approaches by the examining doctors in each check-up. This would explain discrepancies, presented in Table 7, in the frequency of the diseases detected in each examination. That is, the second examination detected a greater proportion of facial and glandular diseases than the more general check-up.

The differences concerning labial and cutaneous diseases are much less pronounced. The second check added new groups of diseases to the list, especially dental diseases (caries and fluorosis). In their summary of this detailed check, the authors of the report emphasised that not all the diseases detected had the same nutritional implications, so the most prevalent diseases did not necessarily have a nutritional base. They even claimed that ‘*the reporting ratio of physical findings of nutritional significance is low*’ [14] (p. 25).

In fact, they only singled out four nutrition-related diseases: angular scars and cheilosis (among the labial diseases), with 28% incidence; red, swollen or receded gums (among the periodontal diseases), with 25% prevalence; and follicular keratosis, which had the greatest incidence (64%).

**Figure 4:**
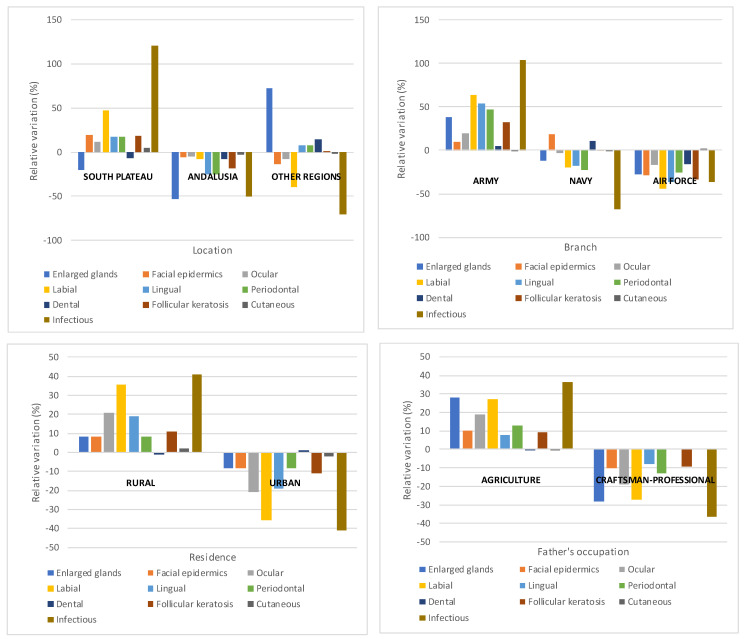
Relative variation of diseases in the detailed medical check-up, according to tabulation variables.

The distribution of these diseases was not homogenous according to location, branch, residence and father’s occupation. Figure 4 (see data Appendix A Table A2) illustrates their deviation from the average, and confirms the diagnostic differences detected in the sample. It is clear that above average prevalence values mostly affected troops deployed in the ‘South plateau’, serving in the army, coming from a rural background and whose fathers worked in agriculture. This is particularly clear with infectious, labial and lingual diseases, and glandular swelling. However, concerning the latter, there is a considerable degree of variation in ‘Other regions’ owing to the results yielded by the army barracks in La Coruña, where goitre was detected in 13% of the subjects. Therefore, the contrast values used for the study of the association between variables suggest that the variables are interdependent (see Table 8); Cramer’s V coefficient results are very modest. As the disaggregation of the contrast values shows, only 3 of the 10 groups of diseases (follicular keratosis and ocular and facial diseases) challenge this conclusion. For these diseases, the number of diagnoses does not seem to have depended on region, place of residence, occupation of the father, branch, and region.

**Figure 5:**
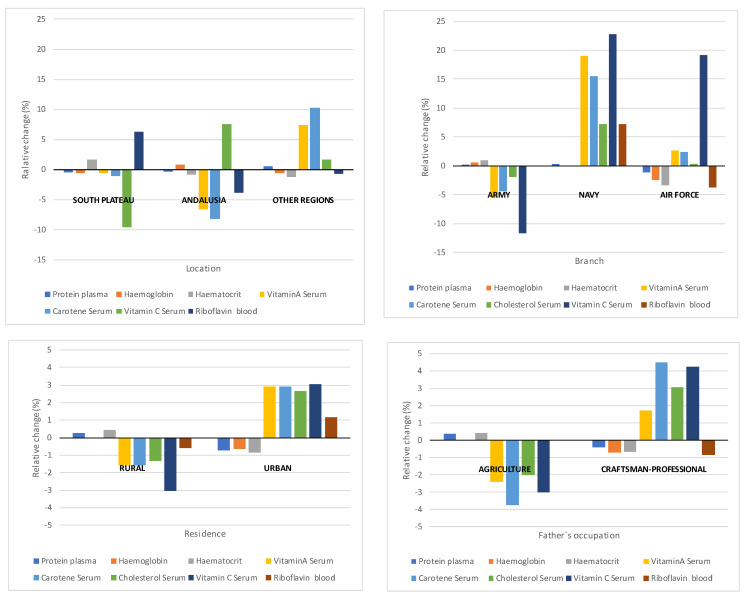
Relative variation of biochemical indicators in blood and urine tests according to tabulation variables.

Blood and urine samples were taken from 520 of the 2145 subjects examined in detail. These samples underwent laboratory tests and the results were assessed according to the criteria set forth in the ICNND manual. Twelve indicators were taken into consideration (nine for the blood samples and three for the urine samples). We shall pay special attention to the former, because they are more directly related to deficiencies in nutrition. The data is presented in Table 9 and Figure 5. This table presents average values, standard deviations, estimated associated intervals, and assessment scales. The results of four indicators (plasma proteins, haemoglobin, haematocrit, and carotene) present average values and acceptable or high variation intervals. Another three (vitamins A and C and riboflavin) present acceptable average levels, but the variation intervals indicate that a significant proportion of the participants presented excessively low values. Finally, concerning cholesterol, both the average and the interval were below acceptable levels. In this instance, the ICNND’s report included no scale of reference, and we use the one published by the WHO. Even so, the document refers to these values as “significantly lower” than those found in the same age group in the USA. If we assume a normal distribution for each of the biomarkers included in the table, we can roughly quantify the percentage of the affected population and conclude that approximately 16% of the sample presented low values, even accounting for a below average standard deviation.

Figure 5 illustrates the relative variation of results from the average value in each item in the analysis, based on the usual tabulations used in the report. The average values increase as we move from south to north (Andalusia, South plateau, and ‘other regions’, in that order). Army recruits presented lower average values than the navy and, especially, air force recruits. Finally, urban recruits whose fathers were craftsmen or professionals present above average values, clearly standing out from recruits from a rural background.

The biochemical analysis sought evidence for both deficiencies that had been previously detected in the medical examinations and those that remained unobserved. Therefore, all three analytical steps must be regarded jointly, and, applying this logic, the authors of the report explored the correlations between four indicators—haemoglobin, riboflavin (in blood and urine), vitamin C, and creatinine—and a series of pathologies detected during the medical examinations and potentially related to low results in these four variables. These pathologies included lingual, labial, facial, periodontal, follicular, and ocular diseases. Even using statistical significance tests, however, no correlation between these variables could be attested, except that concerning low levels of vitamin C and some periodontal symptoms (red and swollen gums).

Summing up, the review of the results of the clinical examinations leads to the following conclusions: (a) High prevalence (above 50%) of 3 of the 10 groups of diseases diagnosed; (b) Among these, only a few were indicative of nutritional deficiencies (4 of 52); (c) Most biochemical indicators yielded acceptable or high average values; however, given the dispersion range of the data, it is assumed that some groups within the analysed sample presented deficient values in some of these indicators, for instance vitamin A and riboflavin; (d) Average prevalence and biochemical values depended on armed forces branch, location of the military facility, place of residence of recruits, and occupation of the father. This was unsurprising, given the selection bias in the sample. In this way, recruits serving in the Army, deployed in the ‘South plateau’ region, from rural backgrounds and with fathers working in agriculture, presented relatively worse biomedical conditions than the rest.

### 3.3. Dietary Survey

As noted in the introduction, the interest to assess the nutritional standards of the military population and their impact in terms of disease led the final part of the report to analyse the diets consumed by recruits in the different military facilities. It was expected that a better understanding of the diet would lead to a more accurate analysis of the medical results and to better-tailored recommendations concerning the drafts’ diet.

Table 10 presents an overall balance of the diet served to the drafts in the twelve military facilities included in the survey. Two values are provided for each nutrient: laboratory analytical results and calculated values, along with standard tables. The analytical results, which were in principle more accurate, are probably less representative, because they used individual rations and menus as reference, while the calculated values were based on the total amount of food entering the barracks’ kitchens, which was counted over a longer time span.

The discrepancies found between both values are significant for some micronutrients, but negligible in terms of overall calorie and protein intake. We shall use the calculated values because it was the value used to calculate the composition of the diet for the whole population, which was shown in the report next to that of the soldiers for comparative purposes. It is inferred that soldiers took more calories, proteins, and other micronutrients than the population in general, except for vitamins A and C. The scale of acceptable levels used by the report, which is reproduced in the final column of Table 10, presents a more accurate perspective on the possible shortcomings of Spanish military diets.

In this way, the deficit of vitamin A in military diets looks more significant, because the intake of this vitamin among the population in general was far below the recommended minimum, so it follows that its presence in military diets was extremely low.

The authors of the report also valued fat intake as ‘fairly low’, although it was above that of the general population, which was far below that of the American population—used as reference by default. This contributes to explaining the surprisingly low cholesterol levels reflected by the results.

The average intake of proteins, riboflavin, and calcium was barely above the minimum recommended levels, even though it was greater than those found among the population in general. As such, the smallest reduction of these nutrients in the diet would send many military drafts below the recommended levels. The variations in the diets consumed by military drafts can be analysed according to two variables: the location of their military facility and armed forces branch. Appendix A Table A3, which presents the average diet in each of the six groups of military facilities, reveals that the main factor of variability was the armed forces branch. Army barracks yield the lowest values, and margin of variability—in terms of vitamin A, riboflavin, and fats—followed by barracks in the southern plateau, in which the presence of this armed forces branch was predominant.

## 4. Discussion

One aspect of the results presented in the previous sections is particularly worthy of comment. It has to do with their relationship with dietary patterns.

The authors of the report explored the relationship between the biochemical markers yielded by blood and urine samples and some diseases. However, they failed to draw a connection between their study of military diets and these diseases. As noted, they were cautious when it came to drawing direct causal links between biochemical indicators and some of the nutritional deficits. The reasons for this were various, including the small size and composition of the sample, and the multi-causal nature of the diseases detected. However, the information provided by clinical exams could be easily combined.

Table 11 presents a list of micronutrients and their association with nutritional deficit-related diseases, according to diagnostic tables published by FAO [32]. The data correspond to the clinical examinations analysed in the previous section and use the same evaluation scales applied by the ICNND. Overall, the table confirms the conclusions of the clinical observations, that is, that the military population did not suffer severe nutritional deficits. Except for vitamin A and riboflavin, micronutrients were found to reach acceptable levels, and in some instances even high levels. However, the clinical examinations detected a series of symptoms compatible with nutritional deficit-related diseases. The proportion of soldiers affected was, in most cases, under 10%, although some diseases, such as hyperthyroidism (goitre), were very unevenly distributed, as the results obtained in La Coruña (9.8%—3 times higher than average) and Badajoz (6.3%) suggest. Both cities are in regions in which this disease was historically endemic [33]. The most widespread diseases, follicular keratosis and nasolabial seborrhea, are related to deficits in vitamin A and riboflavin, respectively. This partially coincides with the results of the assessment of military diets, which found these micronutrients to be served in short supply.

In contrast, the authors of the report emphasised the possible excess in the intake of fluorine, detected through the elevated incidence of fluorosis, which they related to the high content of fluorine in drinking water. According to the authors of the report, this would explain the low incidence of caries and the relatively good dental health of the soldiers.

## 5. Conclusions

This article presents and examines the ICNND’s report on the nutritional standards in the Spanish armed forces in the late 1950s. This report was the largest, in terms of size of sample, of a series of monographs dealing with different national armies. The study diagnosed the nutritional state of the Spanish armed forces and advanced a series of recommendations to improve their organisation in this field. We have examined the report’s data, the three examinations undertaken by the medical team, and, less in detail, the nutritional content of military diets. The report’s conclusion was that ‘… *the nutrition of the Spanish Armed Forces was satisfactory except for the relatively minor problems*’ [14] (p. 103). This statement, supported by the results commented upon in Section 4, must, however, be challenged, at least in part, given the detection of some minor deficiencies in specific territories. Two factors necessarily affect our assessment of the report. First, its representativeness with regard to the whole of the armed forces at the time. Second, its contribution to our understanding of the overall biomedical standards of the adult male population in the 1950s, specifically the cohorts born between 1935 and 1937.

Concerning the first of these factors, the sample was not representative in terms of territorial and armed forces branch distribution. The military population of the northern regions was underrepresented, as was the army. Although it is hard to determine what are the effects of these biases on the results, these would probably be significant, because the nutritional status of recruits depended to a large extent on their region of origin and the armed forces branches they were joining, as the three branches had different access criteria. These differences are clearly illustrated by the clinical examinations of, and nutritional status among, army and air force recruits. Army recruits were shorter, came mostly from rural areas, and were more likely to present nutritional deficit-related diseases, as confirmed by the biochemical indicators. Air force recruits, in contrast, were taller, a greater proportion of them came from urban areas, their parents’ occupations were more varied (including craftsmen, professionals, and others), and their clinical results were better in all counts.

Another factor that needs to be taken into account, as pointed out by the authors of the report, was the positive effect that army diets could have on the nutritional status of recruits, who in many cases were better fed during this period of their lives that they had been before. Therefore, the ‘good health status’ of Spanish soldiers would be, to a large extent, brought about by the institution itself. The scale of this effect depended on the conditions in which the soldier arrived when he joined the ranks, being particularly beneficial for recruits with a low socio-economic status. This would also contribute to reduce inter-personal differences. The consequences of this effect cannot be neglected, although they are nigh impossible to quantify.

Concerning geographical bias, the geographical origin of recruits, already mentioned in the preceding paragraph, must be taken into account. Most recruits in the sample came from the centre and south of the Iberian Peninsula, regions in which living standards were worse than in the rest of the country [29,34]. On the other hand, the number of recruits drafted that, in the event, never served in the armed forces, was relatively high. Military statistics for the 1950s, which were regularly published from 1960 onwards, indicate that in 1958, 30% of all drafts were excluded from the service temporarily or for good for medical and anthropometric reasons and, to a lesser extent, for having family charges. Another 10% never turned up and were declared deserters. The bias introduced by the absence, for one reason or other, of 40% of the male population in military age precludes the possibility of directly extrapolating the results of the survey to the whole of this generation. The report did not take this into consideration, as its main aim was to know the nutritional standards of active soldiers and compare their diets with those of the Spanish population as a whole. As noted, this comparison requires up-to-date data on nutrient consumption and inequality in the late 1950s, and, especially, a more precise understanding of the energy requirements of those serving in the armed forces.

Despite the relevance of these factors, the data published in the report allow us to gauge the scale of the regional differences in several indicators related to nutritional deficits. This, in turn, illustrates social inequality in a very general level. In conclusion, despite the limitations of the SNSAF’s survey, the combination of medical and clinical data and the wealth of biomarkers that it provides turns this hitherto untapped source into an inescapable piece of evidence for the Spanish nutritional transition and its effects on the health status of important sections of the adult population.

## Figures and Tables

**Table 1:** Spain: Nutrition Survey of the Armed Forces. Table of contents.

Item	Contents	Number Tables	Tables in Document
	
Population surveyed (Total sample)	Location, province of birth and other sociodemographic and anthropometric data		
		4	4 to 7
Brief examination	Percent incidence of clinical findings by location, service, father’s occupation, standard weight and examiner		
		5	8 to 12
		
Detailed examination	Percent incidence of clinical findings by location, service, father’s occupation, standard weight and examiner	5	13 to 17
		
		
Biochemical examination	Biochemical findings by location, service, father’s occupation, standard weight and other relations with particular diseases		
		7	18 to 24
		
Dietary survey	Location, nutrient composition and values some Spanish foods		
		3	25 to 27
	Food waste and daily and seasonal nutrient intakes	10	28 to 37
	Comparison with other food intakes and other information about prices of food	6	38 to 43
		

**Table 2:** Distribution of examined soldiers by region of deployment and place of birth.

		Soldiers					
		Subjects in Military Region		Born in the Province		Proportions Born	
Regions	Provinces	(n)	(%)	(n)	(%)	in One of the Three Provinces	in the Province Where They Served
South Plateau	Madrid, Badajoz, Albacete	4340	40.5	2338	21.8	53.9	41.6
Andalusia	Cadiz, Málaga, Granada	3200	29.8	2095	19.5	65.5	38.8
Other regions	La Coruña, Valencia and Mallorca	3187	29.7	1722	16.1	54.0	43.
	Other provinces			4572	42.6		
Total		10,727	100	10,727	100.0	57.4	41.3

**Table 3:** Distribution of subjects by place of birth, draft complements, and population between the ages of 15 to 24 in the 1950 population census (Army regions).

Military	Ranking	Survey	Ranking	Complement	Ranking	Pop(15–24a)
Regions		(%)		(%)		(%)
		(1)		(2)		(3)
Reg 1	1	34.5	1	18.0	1	19.1
Reg 2	5	9.7	2	13.2	2	13.8
Reg 3	4	7.0	3	11.4	3	11.9
Reg 4	7	3.1	5	10.3	4	10-2
Reg 5	10	0.9	9	6.6	9	5-5
Reg 6	9	1.1	4	10.4	5	9.7
Reg 7	8	3.1	6	9.6	7	8.9
Reg 8	3	15.9	7	8.9	6	9.6
Reg 9	2	23.2	8	7.2	8	6.9
General captaincies	6	1.5	10	4.5	10	4.5
Total		100		100		100

Column 1, SNSAF. Column 2, data published by Rodrigo Fernández [22] (pp. 166–167). Column 3, 1950 population census.

**Table 4:** Distribution of survey subjects by armed forces branch (in %) Subjects by military branch.

Regions (1)	Army	Navy	Air Force	3 Branches
South Plateau	36.7	0.0	3.7	
Andalusia	16.8	13.1	0.0	
Other regions	14.9	3.7	11.1	
Total	68.4	16.8	14.8	100.0
Distribution of 1967 draft	84.2	8.8	7.0	100.0

Column 1: South plateau: Madrid (El Goloso and Alcalá de Henares), Badajoz and Albacete. Andalusia: Cádiz, Malaga y Granada. Other regions: La Coruña, Valencia and Palma de Mallorca.

**Table 5:** Characteristics of the soldiers taking part in the survey by region and armed forces branch.

Characteristics	South Plateau	Andalusia	Other Regions	Army	Navy	Air Force	Total
*N*	4340	3200	3187	7340	1800	1587	10,727
Age							
% of soldiers aged 21–22	85.9	69.7	63.2	81.5	59.0	58.8	74.3
Estimated average age	22.1	21.9	22.0	22.2	21.6	21.6	22.0
Time in the services							
% soldiers < 3 months	71.5	55.0	0.2	58.9	30.3	0.2	45.4
% soldiers > 24 months	0.5	6.8	10.2	0.2	14.3	18.2	5.3
Estimated average time in the service	5.0	7.6	12.0	5.8	11.2	13.4	7.9
Type of place of birth							
% Soldiers with a rural background	77.0	67.3	60.1	77.7	52.5	47.9	69.1
Father’s occupation							
Agriculture	63.5	60.3	48.8	65.8	45.4	37.3	58.2
Craftsman and professional	24.0	24.6	31.5	22.2	34.7	36.4	26.4
Others	12.5	15.2	19.8	12.0	20.0	26.3	15.5
Average height (in cm)	164.4	163.9	165.2	164.2	164.4	166.4	164.6
Standard deviation	6.1	5.7	6.3	6.0	5.5	5.7	
Average weight (in kg)	60.6	60.6	63.8	61.2	60.9	63.4	61.5
Standard deviation	6.5	6.5	7.3	6.6	7.2	6.8	

**Table 6:** Differences in anthropometric indicators by region and armed forces branch.

	Height		Weight	
Regions and Branches	Value t	Level-*p*	Value t	Level-*p*
South plateau-Andalusia	3.402	0.001 *	0.03	0.978
Andalusia-Other regions	8.291	0.000 *	18.98	0.000 *
South plateau-Other regions	5.345	0.000 *	20.23	0.000 *
Army-Air force	14.14	0.000 *	11.32	0.000 *
Air force-Navy	10.42	0.000 *	10.29	0.000 *
Army-Navy	1.22	0.223	1.45	0.147

* *p* < 0.01.

**Table 7:** Medical check-ups: incidence of disease per 100 subjects.

	General	Detailed
Disease Group	(n)	(n)
Enlarged glands		4.2
Facial and glandular	14.4	
Scurvy	4.8	
Muscular	1.4	
Facial epidermis		28.9
Ocular		59.3
Labial	33.9	32.4
Lingual	5.5	30.9
Periodontal		30.8
Dental		54.5
Follicular keratosis		63.6
Cutaneous	20.5	27.0
Infectious		8.9

**Table 8:** Detailed medical check-up: Statistical association analysis between variables.

		Association Test		Measurement
Variables	Diseases	Chi-Square	G-Square	Cramer’s V
Residence (Rur/Urb)-Disease		71.01 ***	73.97 ***	0.099
Father’s occupation-Disease		44.83 ***	45.65 ***	0.084
Branch- Disease		134.24 ***	139.73 ***	0.096
Military region-Disease		237.30 ***	243.47 ***	0.127
		Disaggregation diseases Stat- G2		
Variables	Diseases	Facial	Ocular	Labial
		Epidermis		
Residence (Rur/Urb)-Disease		0.00	5.62 *	14.70 ***
Father’s occupation-Disease		1.73	1.39	4.01 *
Branch- Disease		2.53	3.54	33.16
Military region-Disease		27.84 ***	1.21	40.03 ***
		Disaggregation diseases Stat- G2		
Variables	Diseases	Lingual	Periodontal	Dental
Residence (Rur/Urb)-Disease		0.17	6.88 ***	28.93 ***
Father’s occupation-Disease		5.01 *	0.67	17.64 ***
Branch- Disease		10.70 **	5.00 *	31.14 ***
Military region-Disease		16.87 ***	13.68 ***	49.69 ***
		Disaggregation diseases Stat- G2		
Variables	Diseases	Follicular	Cutaneous	Infectious
		keratosis		
Residence (Rur/Urb)-Disease		0.70	5.59 *	11.38 ***
Father’s occupation-Disease		0.92	6.04 *	8.49 **
Branch- Disease		2.71	23.07 ***	28.12 ***
Military region-Disease		2.69	4.81 *	86.71 ***

* *p* < 0.05, ** *p* < 0.01, *** *p* < 0.001.

**Table 9:** Biochemical indicators in blood and urine analyses.

			Interval	+1/−1 Stan. Dev	Assessment ICNND Levels		
Item Analysis	Average	Standard dev.	Min.	Maximum			
					Low	Acceptable	High
Plasma Protein	8.1	0.45	7.61	8.51	6–6.4	6.5–7	>7
Gm/100 mL							
Hemoglobin	15.5	1	14.5	16.5	12–13.9	14–15	>15
Gm/100 mL							
Haematocrit	47.9	3	44.9	50.9	36–41	42–45	>45
Percentage							
Vitamin A Serum	37.7	1.9	35.8	39.6	10–19	20–50	>50
Mcg/100 mL							
Carotene Serum	79.7	26.5	53.2	106.2	20–39	40–100	>100
Mg/100 mL							
Cholesterol Serum	150	35	115	185		180–200 (*)	
Mg/100 mL							
Vitamin C Serum	0.33	0.21	0.12	0.54	0.10–0.19	0.20–0.40	>0.40
Mg/100 mL							
Riboflavin Blood	17.3	4	13.3	21.3	10–14.9	15-19.9	>20 (*)
Mcg/100 mL							

(*) With reference to WHO not ICNND.

**Table 10:** Daily intake of nutrients in military mess halls and the Spanish population as a whole.

				Variation	
Item	Mode	Armed	Spanish Population	Armed Forces/	
		Forces		Spanish Population	ICNND Levels
		Total	1951–1954	(Percentage)	Acceptable
Calories	Estimated	3330	2553	30.4	
	Analysed	3270			
Protein (gm)	Estimated	106	68	55.9	100–150
	Analysed	109			
Fat (gm)	Estimated	87	80	8.8	
	Analysed	73			
Calcium (gm)	Estimated	0.5	0.5	10.2	0.40–0.80
	Analysed	1.0			
Iron (mg)	Estimated	18	11.0	63.6	9–12
	Analysed	72			
Total Vitamin A (I.U)	Estimated	1690	2205	−23.4	3500–5000
	Analysed	2570			
Thiamine (mg)	Estimated	1.5	1.2	25.8	0.30–0.50
	Analysed	1.4			
Riboflavin (mg)	Estimated	1.3	1.0	26.0	1.2–1.5
	Analysed	1.1			
Niacin (mg)	Estimated	15.7	12	30.8	10–15
	Analysed	18.6			
Vitamin C (mg)	Estimated	63	113	−44.2	30–50
	Analysed	70			

**Table 11:** Deficiency-related diseases and associated markers.

Micronutrients	Estimated:	Units	Amount	Assessment	Symptom-Disease	% Soldiers Affected
Vitamin A	Blood test	Mcg/100 mL	37.7	Acceptable	Follicular keratosis	63.6
	Consumed diet	Intern. Unit	1.690	Deficient		
	Raw diet	Intern. Unit	2.570			
Carotene	Blood test	Mg/100 mL	79.7	Acceptable	Bitot’s spots	0.7
Vitamin C	Blood test	Mg/100 mL	0.3	Acceptable	Bleeding and swollen gums	2.1
	Consumed diet	Mg	63.0	High	(Scurvy)	
	Raw diet	Mg	70.0			
Riboflavin	Blood test	Mcg/100 mL	17.3	Acceptable	Labial cheilosis	10.5
					Scrotum Dermatitis	0.3
	Consumed diet	Mg	1.3	Low - Acceptable	Geographic tongue	0.6
	Raw diet	Mg	1.1		Nasolabial seborrhea	25.5
Thiamine	Urine test	Mcg/gm	122.0	Acceptable	Sensitive thighs	0.3
Niacin	Urine test	Mcg/gm	3.5	Acceptable	Atrophied tongue	6.3
	Consumed diet	Mg	15.7	High		
	Raw diet	Mg	18.6			
Proteins	Blood test	Gm/100 mL	8.1	High	Thickness skin folds	20.3
	Consumed diet	Gm	106.0			
	Raw diet	Gm	109.0			
Fluorine	No data				Caries	16.2
					Fluorosis	20.7
Iodine	No data				Enlarged thyroid	3.3

## Data Availability

Data analysed in this work was published in the quoted document: “Spain. Nutrition Survey of the Armed Forces, a report by the Interdepartmental Committee on Nutrition for National Defence 1958”.

## Appendix A

**Table A1:** List of diseases diagnosed in medical check-ups.

Tabulated Diseases	Disease Diagnosed	
Group	Detailed Examination	Brief Examination
Enlarged glands	Thyroid	X
	Parotid	
	Submaxilliary	
Facial epidermis	Nasolabial Seborrhea	X
	Other Seborrhea	
	Erythema	
	Pigmentation	
Ocular	Thickened Conjunctivae	
	Pinguecula	
	Bitot’s Spots	X
	Circumcorneal Injection	
	Conjunctival Injection	
	Blepharitis	
Labial	Angular Lesions	X
	Angular Scars	X
	Cheilosis	X
Lingual	Filiform Atrophy	X
	Fungiform Atrophy	
	Papillary Hypertrophy	
	Furrows	
	Fissures or Erosions	
	Serrations	
	Red, Tip or Margins	
	Magenta Tongue	X
	Geographic Tongue	
Periodontal	Red or swollen	
	Atrophy of Papillae	
	Recession	
	Bleeding or Scorbutic	X
Dental	Caries	
	Edentulous	
	Worn	
	Fluorosis	
	Malposition	
	Anywhere	X
Follicular Keratosis	Arms	
	Back	
	Thighs	
	Buttocks	
	Chest	
Cutaneous	Perifolliculosis	
	Xerosis	
	Acneform Eruption	
	Scrotal Dermatitis	X
	Hyperpigmentation	
Incidence < 0.1% (Not considered)	Loss of Ankle Jerks	X
	Loss of Vibratory Sense	
Skinfold (*)	Arm	
	Scapula	
Infectious	Malaria	
	Typhoid	
	Brucellosis	

(*) Counted as cutaneous disease according to available data. X represents the items mentioned in Brief examination.

**Table A2:** Detailed medical check: incidence of diagnosed diseases by location, armed forces branch, residence and occupation (per 100 subjects).

	Location			Branch				Residence			Father’s Occupation				
Diseases	South Plateau	Andalusia	Other Regions	Average	Army	Navy	Air Force	Average	Rural	Urban	Average	Agriculture	Craftsman- Professional	Average	Total
Enlarged glands	3.48	2.06	7.49	4.34	4.90	3.13	2.58	3.54	4.50	3.80	4.15	4.90	2.74	3.82	4.20
Facial epidermis	33.79	26.91	24.46	28.39	30.40	32.69	19.81	27.63	30.40	25.70	28.05	30.60	25.01	27.81	28.90
Ocular	65.42	56.10	54.41	58.64	64.36	52.49	45.08	53.98	66.60	43.70	55.15	70.11	47.89	59.00	59.30
Labial	45.43	28.62	18.63	30.89	39.88	19.47	13.61	24.32	38.90	18.50	28.70	41.10	23.67	32.38	32.40
Lingual	42.87	21.44	23.92	29.41	37.04	19.81	15.32	24.06	34.20	23.30	28.75	33.90	28.93	31.41	30.91
Periodontal	35.44	22.73	32.44	30.21	36.30	19.18	18.40	24.63	32.40	27.50	29.95	33.30	25.76	29.53	30.82
Dental	51.23	50.83	62.70	54.92	55.79	59.19	45.27	53.41	54.20	55.40	54.80	54.70	54.79	54.74	54.50
Follicular keratosis	73.86	50.81	62.50	62.39	72.13	55.05	36.40	54.53	67.90	54.50	61.20	68.90	57.42	63.16	63.59
Cutaneous	28.63	26.33	26.70	27.22	27.30	27.08	27.91	27.43	27.60	26.50	27.05	27.50	27.79	27.64	26.98
Infectious	17.30	3.89	2.31	7.83	11.63	1.87	3.62	5.71	10.80	4.50	7.65	11.50	5.35	8.43	8.90

**Table A3:** Daily intake of nutrients in military canteens by branch and region.

			Branch				Region			Armed	Spanish
Item	Modality	Army	Air Force	Navy	Variation Range	South	Andalusia	Other	Variation Range	Forces	Population
					Max-Min (%)	Plateau		Regions	Max-Min (%)	Total	1951–1954
Calories	Estimated	3449.8	3316.2	3159.3	9.2	3406.7	3321.6	3406.3	2.6	3330.0	2553.0
	Analysed	3442.9	3223.8	3115.8	10.5	3222.4	3441.6	3349.9	6.8	3270.0	
Protein (gm)	Estimated	108.0	102.7	114.4	11.4	105.2	112.7	104.7	7.7	106.0	68.0
	Analysed	115.1	105.0	112.8	9.6	105.7	122.0	105.4	15.8	109.0	
Fat (gm)	Estimated	83.5	97.4	89.4	16.6	87.4	83.2	97.5	17.2	87.0	80.0
	Analysed	76.0	69.9	60.6	25.4	80.4	64.2	72.0	25.2	73.0	
Calcium (gm)	Estimated	0.5	0.6	0.6	10.2	0.5	0.5	0.5	5.8	0.5	0.5
	Analysed	1.1	1.1	1.0	7.0	1.1	1.1	0.9	25.4	1.0	
Iron (mg)	Estimated	19.1	17.4	20.0	14.6	17.9	20.3	18.3	10.9	18.0	11.0
	Analysed	77.4	61.0	70.3	26.9	70.4	77.2	69.8	10.7	72.0	
Total Vitamin A (I.U)	Estimated	1293.9	2797.8	2213.0	116.2	1416.8	1976.2	1714.5	39.5	1690.0	2205.0
	Analysed	1904.5	2901.2	3271.4	52.3	2227.1	2466.1	2291.1	10.7	2570.0	
Thiamine (mg)	Estimated	1.7	1.4	1.4	17.9	1.6	1.6	1.5	5.5	1.5	1.2
	Analysed	1.5	1.3	1.6	21.2	1.5	1.6	1.1	43.2	1.4	
Riboflavin (mg)	Estimated	1.2	1.3	1.5	25.3	1.2	1.4	1.3	16.8	1.3	1.0
	Analysed	1.0	1.1	1.1	9.6	1.0	1.1	1.2	23.7	1.1	
Niacin (mg)	Estimated	16.2	15.7	14.3	13.7	15.1	15.6	18.0	19.2	15.7	12.0
	Analysed	20.3	17.3	19.0	17.4	16.0	22.9	18.7	43.9	18.6	
Vitamin C (mg)	Estimated	62.1	58.7	51.6	20.3	55.4	57.1	78.3	41.3	63.0	113.0
	Analysed	67.9	75.2	73.1	10.6	52.8	81.9	82.1	55.1	70.0	
